# Does the reticulospinal tract mediate adaptation to resistance training in humans?

**DOI:** 10.1152/japplphysiol.00264.2021

**Published:** 2022-07-14

**Authors:** Elliott Atkinson, Jakob Škarabot, Paul Ansdell, Stuart Goodall, Glyn Howatson, Kevin Thomas

**Affiliations:** ^1^Department of Sport, Exercise and Rehabilitation, Faculty of Health and Life Sciences, Northumbria University, Newcastle-upon-Tyne, United Kingdom; ^2^School of Sport, Exercise and Health Sciences, Loughborough University, Leicestershire, United Kingdom

**Keywords:** neuromuscular, StartReact, strength training, TMS, TES

## Abstract

Resistance training increases volitional force-producing capacity, and it is widely accepted that such an increase is partly underpinned by adaptations in the central nervous system, particularly in the early phases of training. Despite this, the neural substrate(s) responsible for mediating adaptation remains largely unknown. Most studies have focused on the corticospinal tract, the main descending pathway controlling movement in humans, with equivocal findings. It is possible that neural adaptation to resistance training is mediated by other structures; one such candidate is the reticulospinal tract. The aim of this narrative mini-review is to articulate the potential of the reticulospinal tract to underpin adaptations in muscle strength. Specifically, we *1*) discuss why the structure and function of the reticulospinal tract implicate it as a potential site for adaptation; *2*) review the animal and human literature that supports the idea of the reticulospinal tract as an important neural substrate underpinning adaptation to resistance training; and *3*) examine the potential methodological options to assess the reticulospinal tract in humans.

## INTRODUCTION

Resistance training is commonly used to increase muscular strength in humans. Prolonged resistance training is accompanied by changes in muscle structure; however, early increases in force production have been proposed to be predominantly underpinned by neural adaptations, as detectable structural changes to the muscle are modest or absent in the initial phases of training (1). Although this supposition is widely accepted and supported by experimental data (2, 3), the neural systems underpinning increased strength as a consequence of resistance training in humans are unclear (4). Previous work in humans has primarily focused on corticospinal tract (CST) adaptations, with equivocal outcomes (5, 6). It is therefore possible that other neural adaptations might play a role in mediating increases in muscle strength.

The reticulospinal tract (RST) is a bilateral, descending pathway integral to both gross motor function and forceful movements (7, 8). In contrast to the CST, the RST has been rarely investigated in humans, most likely because its location in the brain stem makes noninvasive stimulation challenging. Despite these difficulties, emerging evidence has indicated that the RST might be a significant contributor to the neural adaptations to resistance training (9–14). In this mini-review, we *1*) discuss why the structure and function of the reticulospinal tract implicate it as a potential site for adaptation; *2*) review the animal and human literature that supports the idea of the reticulospinal tract as an important neural substrate underpinning adaptation to resistance training; and *3*) examine the potential methodological options to assess the reticulospinal tract in humans.

## ANATOMY AND FUNCTION OF THE RETICULOSPINAL TRACT

The RST is a major descending tract of the spinal cord, and its anatomical structure supports a putative role in resistance training adaptation. The RST consists of multiple fibers originating from the reticular formation, with those of the medial pontine-medullary reticular formation primarily involved in motor control. Nuclei within the medial pontine-medullary reticular formation give rise to a complex array of reticulospinal fibers that can be subdivided further into two generalized tracts, the medial and lateral RSTs (15). Both medial and lateral reticulospinal tracts continue to descend bilaterally terminating at sites throughout the spinal cord, forming monosynaptic connections to motoneurons innervating ipsilateral muscles and polysynaptic connections (via interneurons) to motoneurons with inputs to contralateral muscles (7, 16). This distribution of RST spinal axons allows for bilateral innervation of axial and appendicular muscles (16), alongside synergistic control over extensor and flexor limb muscles (17, 18). In addition, the postsynaptic connections of the RST are highly divergent and innervate many motor unit pools, allowing for the coordination of multiple muscle groups related to gross motor function (13, 18). These neuroanatomical features explain why the RST is a major contributor to postural control and locomotion (8).

In the CST, most neurons descend through the spinal cord contralaterally, with a small number descending ipsilaterally (19). The CST neurons innervate spinal motoneurons through both mono- and polysynaptic connections. Studies in nonhuman primates have shown a greater distribution of polysynaptic than monosynaptic connections (∼80% and ∼20%, respectively) (20). In humans, large polysynaptic CST contributions have been observed to the motoneurons of the forearm (21), upper limb (22), and thigh muscles (23), but it remains unknown whether humans exhibit a similar mono- and polysynaptic CST distribution to that of nonhuman primates (20). What is clear is the human CST is the most advanced among primates because of its capacity for fine motor control (24). Contrastingly to the RST, the contributions of mono- and polysynaptic connections in the CST appear to be the greatest within smaller distal limb muscles, with the diversity of connections supporting fine motor control, such as fractioned finger movements (25, 26). Teleologically, the anatomical structure of the RST is well suited to facilitate the execution of forceful movements, in comparison to the fine motor control mediated primarily by the CST.

This overview of RST and CST anatomy highlights how these neural pathways might contribute to both fine and gross motor function; however, the paucity of research means evidence of such contributions remains equivocal. Despite this, some inferences can be made from existing data. For example, Riddle et al. (27) in their study examined the differences in RST and CST collaterals to intrinsic hand muscles of nonhuman primates. It was observed that projection densities were similar, although CST connections were primarily monosynaptic, whereas those of the RSTs were primarily polysynaptic (27); typically representative of direct and indirect connections to motoneurons, respectively (15). Furthermore, the RST polysynaptic motor evoked potential (MEP) amplitudes were also found to be five times lower than the monosynaptic CST connections (27). This potentially indicates that, despite the RST and CST having comparable projection densities, their distinct connections differentiate their primary roles in gross (RST) compared with fine (CST) motor control. Indeed, MEPs elicited by transcranial magnetic stimulation (TMS) in upper (28) and lower (28, 29) limb muscles in humans potentially support this proposition. It was observed that smaller distal muscles [e.g., first dorsal interosseous (FDI) and tibialis anterior (TA)] displayed larger MEP responses compared with the larger proximal muscles (e.g., biceps brachii and quadriceps) (29). This apparent difference in MEP responses from CST stimulation between smaller and larger muscle groups possibly indicates a difference in the relative role of the RST and CST in motor control, with the lower CST responses in larger proximal muscles potentially indicating greater RST input, and vice versa for the smaller distal muscles. Interestingly, however, the soleus and medial gastrocnemius (MG) display smaller MEPs in response to stimulation of the primary motor cortex (M1), a response atypical of smaller distal muscles and contrary to those of the TA (29), though it is possible that these divergent responses are related to the MG and soleus’ role in postural control and locomotion (8). Although these differences in MEP responses across muscle groups in humans (28, 29) potentially parallel Riddle et al. (27) observations in nonhuman primates, these conclusions remain predominantly speculative and require further research. Despite this, and taken together, it could be hypothesized that it is connection type and strength, not projection density that determines the primary input of the RST and CST to certain muscle groups dependent upon their function.

A potential alternative methodology for examining RST collaterals to various muscle groups is using neck rotations. This action, known as the asymmetric tonic neck reflex, activates cervical afferents, modulating the reticulo-propriospinal pathway and facilitating the RST and the resulting MEP response to TMS (19, 30, 31). McCambridge et al. (32) in their study applied this method in the upper limbs, finding the late portion of the MEP was attenuated in response to TMS in the proximal, but not distal muscles of the upper limb due to neck rotations. This modulation to the late portion of the elicited MEP has been attributed to RST facilitation (32, 33). Although these findings are not definitive, they provide further evidence that proximal muscles involved in gross motor functions receive greater input from the RST.

## EVIDENCE OF RETICULOSPINAL TRACT PLASTICITY IN NONHUMAN PRIMATES

### Selective Descending Tract Lesioning

Invasive experiments in nonhuman primates have been performed to examine how the RST mediates motor function, illustrating how the RST could be an important site of adaption to resistance training in humans. Evidence for RST involvement in gross motor function was first demonstrated by Lawrence and Kuypers, who surgically lesioned the CST (34) and RST (35) of macaque monkeys. After the CST lesion, gross motor function was unaffected, but fine motor function was lost (34). After a period of recovery, the effects of a second lesion on either the RST or rubrospinal tract were assessed in two separate groups. The second lesion of the RST was found to impair gross motor function, whereas the second lesion to the rubrospinal tract impaired fine motor control (35). After a period of recovery after the second lesion of the RST, the affected monkey’s gross motor function eventually recovered (35). This recovery after the RST lesion was attributed to the reorganization of the rubrospinal tract, which is much more prominent throughout the spinal cord in nonhuman primates (7, 36). These studies provide support for the important role the RST plays in gross, forceful movements.

Further research involving direct stimulation of the CST and RST following CST lesioning in nonhuman primates also demonstrates the plasticity of the RST and its putative role in restoring gross motor function. Zaaimi et al. (37) in their study performed contralateral pyramidal lesioning followed by ipsilateral pyramidal stimulations in nonhuman primates, observing weak responses in the forearm and hand, and no return of hand function. Comparatively, direct reticular formation stimulation showed increased mono- and disynaptic postsynaptic amplitudes of forearm flexors postrecovery. These increased postsynaptic amplitudes could reflect the strengthening of RST connections to both motoneurons and spinal interneurons to retain a degree of motor function; with strengthening of the disynaptic spinal interneuronal pathway being indicative of increased bilateral input (37). This could also indicate the mechanistic underpinnings by which gross motor function and grip strength were retained following CST lesioning in the macaque monkeys studied by Lawrence and Kuypers (34). Collectively, findings in nonhuman primates indicate recovery following CST lesioning is concurrent with both increased postsynaptic amplitudes and efficacy of reticulospinal projections, thereby allowing a degree of motor function to be restored.

### Resistance Training

**Figure 1.**
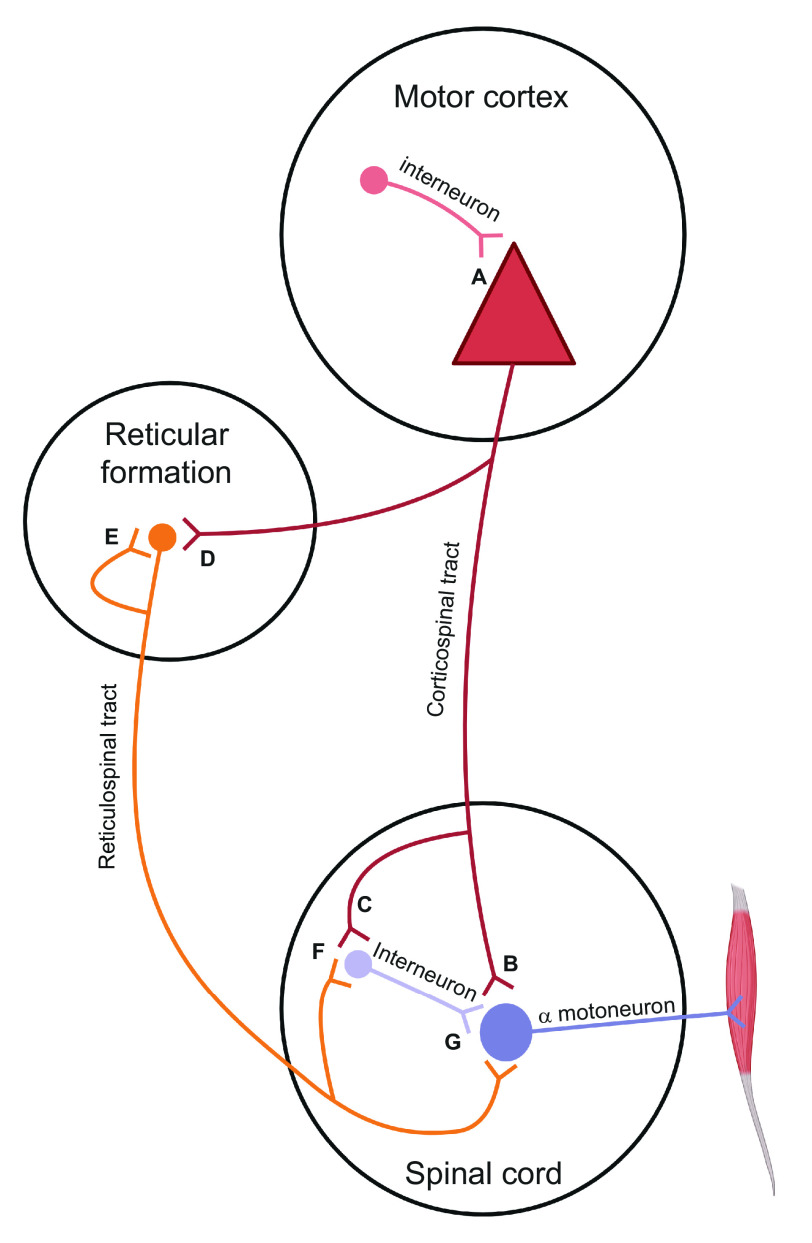
Simplified schematic of corticospinal and reticulospinal pathways to motoneurons potentially contributing to augmentation of muscle force after resistance training. Corticospinal drive to motoneurons could be augmented via downregulation of inhibitory interneurons to the primary motor cortex (*A*), or upregulation of synaptic activity at the mono- (*B*) or disynaptic (via spinal interneuron) connection to motoneurons (*C*). Reticulospinal drive could be augmented via an upregulated cortico-reticular synapse (*D*), reciprocal reticular connection (*E*), and/or disynaptic (via spinal interneurons) (*F*), and monosynaptic (*G*) connections to the α motoneuron. Adapted from Glover and Baker (9). Created with BioRender.com with permission.

To date, the strongest evidence (9) supporting a role for the RST in mediating strength adaptation comes from a single study that directly measured the effects of resistance training on the CST and RST in nonhuman primates. Glover and Baker (9) in their study provided new insight into the underpinning neural adaptions to resistance training. Two female macaque monkeys completed an 8- to 9-wk period of progressive resistance training, a 2-wk washout period, then a further 12 wk of training. The responses to direct M1, CST, and RST stimulation were examined pre- and posttraining. The authors reported no change in CST amplitudes, whereas M1 and RST responses both increased. The increased RST amplitudes were attributed to stronger mono- (Fig. 1*G*) and disynaptic (Fig. 1*F*) connections, resulting in increased synaptic efficacy. The same synaptic strengthening was previously observed following CST lesion in nonhuman primates, potentially as an adaption to the lesion (37). Furthermore, Glover and Baker (9) in their study also observed stronger reticular-reticular (Fig. 1*E*) but decreased cortico-reticular connections (Fig. 1*D*) after resistance training. The role of other neural structures such as Ib spinal interneurons, which the RST has an inhibitory effect on (38), or potential input from muscle spindle afferents cannot also be ruled out for increasing strength. This notwithstanding, the findings of Glover and Baker (9) suggest that the RST is a strong contributor to neural adaption following resistance training in nonhuman primates. Whether a similar mechanism exists in humans is unknown.

## MEASURING RETICULOSPINAL TRACT FUNCTION IN HUMANS

**Figure 2.**
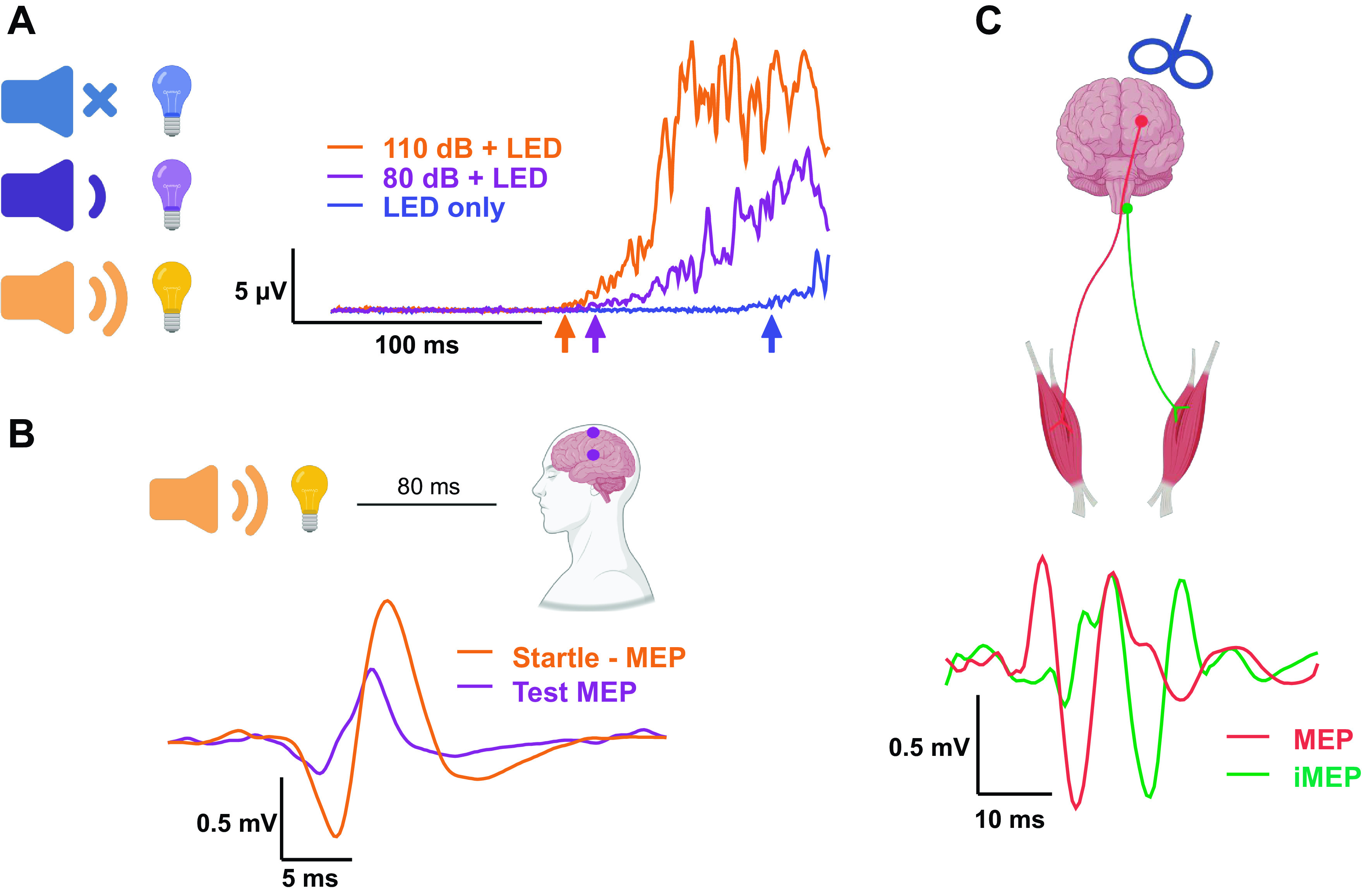
Methodological approaches to probe reticulospinal tract in humans. *A*: StartReact paradigm involves quantifying the reaction time measured in electromyographic activity of muscle in response to a visual cue (visual reaction time, VRT; blue upward arrow), which can be additionally preceded by an auditory stimulus. The startling auditory stimulus (>110 dB) is thought to preactivate reticular pathways resulting in the greater shortening of the reaction time (visual-startling reaction time, VSRT; orange upward arrow) compared with auditory facilitation (80 dB; visual-auditory reaction time, VART; violet upward arrow). Reticulospinal gain is then quantified as the ratio of the difference between VRT and VSRT and between VRT and VART (13). It is hypothesized that the reticulospinal gain would increase with resistance training. Traces are from the personal archive of E. Atkinson (unpublished data) and show an average of 20 responses of the quadriceps femoris muscle. *B*: when a startling auditory stimulus precedes transcranial electrical stimulation (TES) of the motor cortex by 80 ms, the responses (motor-evoked potentials, MEPs) are facilitated compared with when TES is delivered alone (test MEP). The facilitated response is thought to reflect facilitated subcortical structures, likely mediated via the reticulospinal tract (39, 40). It is hypothesized that resistance training would augment facilitation of MEP response to a startling auditory stimulus. Traces are from the personal archive of E. Atkinson (unpublished data) and show an average of five responses of the first dorsal interosseus muscle. *C*: ipsilateral motor evoked potentials (iMEPs) in response to transcranial magnetic stimulation of the motor cortex are thought to represent activation of the reticulospinal tract through the corticoreticulospinal pathway (12). Note the difference in latency between MEPs and iMEPs (∼10.5 vs. 16.5 ms). It remains unknown whether the startling auditory stimulus would cause a similar facilitation of iMEP that is observed with responses to TES. It is hypothesized that iMEPs would increase following a period of resistance training. Traces are from the personal archive of E. Atkinson (unpublished data) and show an average of four responses of the biceps brachii muscle. The data collected for the unpublished traces were from members of the research team during pilot testing in the labs who gave written informed consent. The study was approved by the Northumbria University Faculty of Health and Life Sciences ethics committee (Submission ID: 21321). Created with BioRender.com with permission.

Emerging methodologies give researchers the possibility of bridging the knowledge gap resulting from the inability to directly stimulate the human RST (see Fig. 2 for summary). The singular use of these indirect testing paradigms makes drawing definitive conclusions difficult, but used collectively, they could provide a method to elucidate changes within the RST.

### StartReact

The “StartReact” paradigm quantifies the ergogenic effect of a startling auditory stimulus (SAS, >110 dB, Fig. 2*A*) on reaction time, as an indirect measure of RST function using the startle reflex (13). The startle reflex is a primitive response present in humans following a sudden loud sound (13). When a SAS is imposed during a reaction task, response times are shortened. The shortened response time is a consequence of an involuntary release of a planned movement (41). It is thought that this response originates subcortically, denoting a preactivation of neural pathways (42, 43), hypothesized to be the RST (43).

### Auditory Startle Paired with Transcranial Magnetic and Electrical Stimulation and Electrical Cervicomedullary Stimulation

Transcranial electrical stimulation (TES) and TMS have been paired with SAS to study the contribution of the RST to the evoked electrical response measured in the muscle. These studies (39, 40) found that when a SAS precedes TES of the motor cortex by 80 ms, the MEP response in FDI is facilitated compared with when TES is delivered alone (Fig. 2*B*). The increased MEP response is proposed to be a consequence of an increase in spinal facilitation, probably caused by activation of the reticular formation by the SAS (43). This supposition is supported in human studies that observed the H-reflex (an index of spinal excitability) was enhanced by SAS at 80 ms interstimulus interval (ISI) in the gastrocnemius at rest (44), and in animal studies where direct recordings of reticular neuronal cells show temporal facilitation when exposed to SAS (45). It might also be possible to use electrical cervicomedullary stimulations paired with SAS to elicit similar MEP facilitation to startle-TES (14, 46). In contrast, when SAS precedes TMS of the motor cortex, the MEP response is inhibited at short ISIs (20–60 ms), and no facilitation is observed at 80 ms, despite the aforementioned increase in spinal excitability as a consequence of the SAS (39). The early inhibition with TMS at ISIs delivered <60 ms with respect to SAS was attributed to a suppression in cortical excitability, and the lack of late facilitation at 80 ms ISI was speculated to be a consequence of persistent cortical inhibition canceling the spinal facilitation induced by SAS (39). If the CST or RST adapts to a period of resistance training, it is expected that TMS and TES or cervicomedullary MEP responses will be altered when conditioned with SAS. Specifically, an increase in MEP amplitudes with SAS might signify an adaptation in RST function, or a reduction in cortical inhibition, after training.

### Ipsilateral Cortical Magnetic Stimulation

Indirect RST activation is achievable through delivering magnetic stimulations to the M1, ipsilateral to the target limb. Magnetic stimulation is hypothesized to act on the RST indirectly via the cortico-reticulospinal pathway, resulting in ipsilateral motor evoked potentials (iMEPs) due to the RST’s bilateral structure (7, 12, 18). This could potentially be used to assess changes in RST efficacy following a period of resistance training. Although limited among healthy populations, evidence in clinical populations (stroke patients) provide some support for this proposition. Specifically, patients with stroke display enhanced iMEPs in limbs contralateral to the lesioned hemisphere compared with healthy participants after a period of recovery (12). The enhanced iMEP response could be indicative of a compensatory strengthening of the RST poststroke to preserve various motor functions, an observation similar to previous work in nonhuman primates (35). This adaptation in RST function might also take place in healthy populations after a period of resistance training due to the RST’s involvement in gross motor function (8), a proposition that has yet to be tested. It should be also noted that any change in iMEP amplitude might also be mediated by changes in cortical excitability (39), and thus studying the iMEP response in isolation would not allow for a definitive conclusion on the locus of change.

A challenge in studying iMEPs is the difficulty in which they are elicited in healthy participants (12). One way to potentially overcome this challenge is to assess ipsilateral responses during high force contractions (47) where iMEPs seem easier to elicit, possibly because of a higher ipsilateral activation during high compared with low force tasks. Most recently, quantification of RST function in the upper limbs of healthy participants has been attempted using ipsilateral TMS (48). Although iMEP assessment proved to be successful in older participants, it was not as successful in identifying RST function in younger participants (48). This was potentially due to the use of a standardized 12-kg row that might not have been enough resistance to elicit the high forces necessary to evoke iMEPs, particularly in young participants who are likely to have greater levels of strength (47). Conceptually, resistance training might result in increased iMEP responses, indicating RST plasticity and stronger motoneuronal connections. In addition, because of the known RST activation by SAS (13), it could be hypothesized that iMEP responses are further enhanced when paired with SAS in healthy individuals. There is a risk of cross-hemispheric stimulation when trying to elicit iMEPs through magnetic stimulation (49), though iMEPs are identifiable through longer latencies (Fig. 2*C*) compared with MEPs (50). Therefore, the monitoring of each response is crucial to inform on RST function. Overall, there is evidence that iMEPs might offer an indirect assessment of RST function, and that SAS might enhance the response; however, there are significant challenges in eliciting such responses in healthy individuals.

## EVIDENCE THAT THE RETICULOSPINAL TRACT MIGHT UNDERGO ADAPTION TO RESISTANCE TRAINING IN HUMANS

Clinical populations, such as patients with stroke and spinal cord injury (SCI), are useful models for understanding the role of the human RST in gross motor function. As a progression from the invasive nonhuman primate studies, these clinical populations provide a point of comparison to link observations and demonstrate the potential suitability of the human RST to adapt to resistance training.

### Patients with Stroke

A stroke causes lesions of CST fibers at the cortical level (51); effectively mimicking, to some extent, cortical lesion studies in nonhuman primates (34, 37). Because of its bilateral structure, it is hypothesized that RST efficacy increases poststroke to compensate for the lesioned CST (52). In support, Alagona et al. (12) in their study were able to consistently elicit iMEPs at rest or during weak contractions in patients with stroke, whereas contractions >50% of maximum strength were required to elicit an iMEP in healthy participants (12). Furthermore, an increased ipsilateral hemisphere activation during motor tasks in patients with stroke has been demonstrated with functional magnetic resonance imaging, potentially signifying a strengthening of RST connections to preserve motor function and compensate for the contralateral lesion (53). These findings suggest, such as lesion observations in nonhuman primates, that the human RST undergoes compensatory adaptions poststroke to preserve various motor functions. In nonhuman primates, this recovery is further supported by the existence of a much more extensive rubrospinal tract, which could potentially undergo compensatory adaption alongside the RST (7, 35). This plasticity highlights the possibility for the RST to be a potential site for adaptation to resistance training that prioritizes the training of gross, forceful movements.

### Spinal Cord Injury

Recently, StartReact has been used to quantify the RST function in the FDI of patients with SCI. Responses in the FDI were recorded while participants held an object during fine (pinching of thumb and index finger) and gross (full grip with all fingers) motor tasks. Reaction times to various visual and auditory stimuli were compared between the two tasks (13). It was found that the reaction times of patients with SCI were only reduced during a high force task (which theoretically has strong RST input), whereas in healthy controls reaction times decreased across both high and low force tasks (13, 54). Recent work by Sangari and Perez (14) used the StartReact protocol, with cervicomedullary evoked potentials (CMEPs) paired with SAS, to study evoked responses in the biceps brachii. It was found that patients with SCI had comparable StartReact responses to controls, but CMEP responses paired with SAS were greater (14). Although limited to the upper limbs, these findings indicate that RST neuroplasticity, and greater RST input, compensates for spinal cord lesion to partly restore motor function in the larger upper limb muscles. Aligned to the findings of Baker and Perez (13) and other observations in nonhuman primates (34), RST plasticity in response to CST lesion to retain motor function highlights the RST as a potential site for adaption to resistance training.

## LIMITATIONS OF RESISTANCE TRAINING LITERATURE IN HUMANS

Researchers have attempted to elucidate the neural substrate(s) underpinning human adaptation to resistance training by studying the CST, with equivocal conclusions (55, 56). Recent reviews (6, 57) of CST neuroplasticity following resistance training have found it to be highly variable showing increased, none, or even negative changes to CST amplitudes (6). In addition, a systematic review by Kidgell et al. (5) describes that the overall effect of resistance training on CST MEP amplitudes as “borderline.” Furthermore, a breakdown of the literature suggests the majority of the positive CST observations following resistance training are predominantly in the small distal muscles of the upper limbs, including wrist muscles (58–62), and intrinsic finger muscles (63, 64), alongside similar responses in the small distal muscles of the lower limbs, such as the TA (65–67). Although it has also been reported that the TA shows no change after training (68). The response in larger proximal muscles is inconsistent. Some studies reported positive CST changes in biceps brachii (61) and rectus femoris (69, 70). Conversely, other studies have shown no CST changes in larger proximal muscles, including the vastus lateralis (71), biceps brachii (72), and rectus femoris (73). Interestingly, whereas Beck et al. (67) did observe increased CST excitability in the TA, no change was found in the soleus; a response also observed by Palmer et al. (74) and similar to the evoked TMS responses in the TA and soleus by Brouwer and Ashby (29) with MEPs being greater in the TA compared with the soleus. These contrasting responses between the TA and soleus could reflect the difference in the predominant neural input to these muscles, with a preference for CST control of the TA, and RST for the soleus. Collectively, these findings show an equivocal body of evidence for the CST as a primary site for adaption to resistance training.

The variability in observations and equivocal nature of the literature regarding CST adaptation to resistance training could be attributed to several common confounding issues. For example, the focus on smaller distal muscles potentially biases observations in favor of the CST being the primary site of adaption following resistance training, particularly given the aforementioned preferential CST input to such muscles (27–29). Resistance training paradigms studying small, distal muscle groups lack ecological validity when compared with the typical whole body, gross movements required to increase functional muscle strength. A further cause of these equivocal observations could possibly be the result of frequently using untrained participants and external auditory pacing among the current literature (5, 6, 70). The use of untrained participants could confound observations as exposure to new movement patterns might constitute a form of skill acquisition, with any adaption in CST function possibly being attributed to skill development (75), an acknowledged proposition (70). Therefore, adaptation of CST function after a period of resistance training, and associated increases of strength in untrained individuals, might be better attributed to the learning and improvement of a new movement pattern and a more efficient use of available strength, rather than a transferable increase in muscle strength per se. In fact, one study provides possible evidence of this in which recreationally trained individuals, only performing lower-body resistance training once per week, were recruited to perform a period of squat training (71). It was found that despite significant increases in the strength of their back squat, no subsequent changes in CST excitability were observed potentially signifying those neural changes must be occurring in other pathways, such as the RST. The use of external pacing could confound observations further through introducing greater movement complexity and peripheral feedback (76), thereby increasing the skill requirement resulting in greater CST involvement and subsequent adaptation. It is possible that much of the current literature is unintentionally biased to observing positive changes in CST excitability in response to periods of resistance training, which could explain why there are equivocal reports regarding adaptation.

## SUMMARY AND FUTURE DIRECTIONS

It is evident from the available human literature that the RST remains an understudied potential site of neural adaptation in response to resistance training, primarily because of the inability to noninvasively directly stimulate the human RST. Despite this, some indirect testing methodologies appear promising in elucidating RST function in healthy humans. The lesioning of descending tracts in animal studies has provided invaluable insight into the motor functions mediated by the RST, providing a foundation that gross motor function and the ability to generate high force is mediated by the RST. These observations in lower primates are indirectly replicated in human functional recovery studies of patients with stroke and SCI which emulate, to a limited degree, the lesioning of cortical and spinal structures in animal studies. Perhaps of most relevance, adaptations in RST function, made from direct recordings, primarily explain resistance exercise induced increases in strength in nonhuman primates (9). This finding raises the distinct possibility that a similar adaptation might exist in humans. Future research investigating the neural adaptations to resistance training would be well served by a focus on both CST and RST function, to better understand the neural adaptation underpinning increases in strength of humans. Such research could have significant implications for optimizing resistance training programs for athletic, patient, and healthy populations, and could provoke a conceptual shift in the way practitioners design and implement resistance training to improve muscular strength.

## DISCLOSURES

No conflicts of interest, financial or otherwise, are declared by the authors.

## AUTHOR CONTRIBUTIONS

E.A., J. Š., P.A., S.G., G.H., and K.T. conceived and designed research; E.A. and J. Š. prepared figures; E.A. and K.T. drafted manuscript; E.A., J. Š., P.A., S.G., G.H., and K.T. edited and revised manuscript; E.A., J. Š., P.A., S.G., G.H., and K.T. approved final version of manuscript.
